# Ischämische Hirninfarkte bei Coronavirus-19-Erkrankung

**DOI:** 10.1007/s00108-021-01110-0

**Published:** 2021-08-02

**Authors:** Paulina Laura Sander, Ulrich Cleff, Simon Larrossa-Lombardi, Hatem Ali, Panagiotis Fotiadis, Gernot Reimann, Stefan Rohde, Ulrich Hofstadt-van Oy

**Affiliations:** 1 Klinik für Neurologie, Knappschaftskrankenhaus Dortmund – Klinikum Westfalen, Am Knappschaftskrankenhaus 1, 44309 Dortmund, Deutschland; 2 Klinik für Pneumologie, Intensivmedizin und Schlafmedizin, Knappschaftskrankenhaus Dortmund – Klinikum Westfalen, Dortmund, Deutschland; 3 Klinik für Radiologie, Knappschaftskrankenhaus Dortmund – Klinikum Westfalen, Dortmund, Deutschland; 4 Klinik für Neurologie, Klinikum Dortmund, Dortmund, Deutschland; 5 Klinik für Radiologie und Neuroradiologie, Klinikum Dortmund, Dortmund, Deutschland; 6 Fakultät für Gesundheit, Department für Humanmedizin, Universität Witten/Herdecke, Witten, Deutschland

**Keywords:** Gefäßverschluss, Embolie, Koagulopathie, Thrombolysetherapie, Thrombektomie, Large vessel occlusion, Embolism, Coagulopathy, Thrombolytic therapy, Thrombectomy

## Abstract

Zwei Fälle von Patienten mit der Coronavirus-19-Erkrankung (COVID-19) werden berichtet, bei denen Verschlüsse großer Hirnarterien vorlagen. Diese traten bei einer Patientin in der Früh- als auch im 2. Fall in der Spätphase der COVID-19 auf. Eine Patientin konnte erfolgreich mithilfe der i.v.-Thrombolyse und mechanischer Thrombektomie behandelt werden. Gerinnungsstörungen im Rahmen der COVID-19 können auch bei jüngeren Patienten zu fulminanten Hirninfarkten mit schlechtem Outcome führen. Bezüglich der Ätiologie dieser Gefäßverschlüsse (COVID-19-induzierte Hyperkoagulopathie, Kardiomyopathie, Vaskulitis) besteht weiterer Forschungsbedarf.

## Abstract (en)

This article reports two cases of patients with coronavirus disease 2019 (COVID-19) in which occlusion of large cerebral arteries occurred. These occurred in a female patient in the early stage of COVID-19 and in the second case in the late stage. One female patient could be successfully treated with i.v. thrombolysis and mechanical thrombectomy. Coagulopathy in the course of COVID-19 can result in severe stroke with poor outcome even in younger patients. With respect to the etiology of arterial occlusions (COVID-19-induced hypercoagulopathy, cardiomyopathy, vasculitis) there is a necessity for further research.

Sowohl in der Früh- als auch in der Spätphase der durch das „Schwere-akute-respiratorische-Atemwegssyndrom-Coronavirus Typ 2“ (SARS-CoV2) ausgelösten Coronavirus-2019-Erkrankung (COVID-19) kann bei jüngeren Patienten ein zerebraler Gefäßverschluss auftreten [4, 6, 7]. Im Folgenden werden 2 dieser Fälle vorgestellt.

## Fall 1

### Anamnese

Eine 62-jährige adipöse Patientin mit medikamentös behandelter arterieller Hypertonie wird vom Rettungsdienst wegen plötzlich aufgetretener verwaschener Sprache mit Wortfindungsstörungen vorgestellt; weitere Gefäßrisikofaktoren liegen nicht vor. Zudem bestünde seit etwa 10 Tagen ein Geschmacksverlust; mehrere Arbeitskollegen seien bereits positiv auf das SARS-CoV‑2 getestet worden; respiratorische Beschwerden wurden verneint.

### Klinischer Befund

In der klinisch-neurologischen Untersuchung werden bei der Patientin eine nichtflüssige Aphasie, Dysarthrie sowie rechtsseitige faziale Parese (4 Punkte nach der National Institute of Health Stroke Scale [NIHSS]) diagnostiziert. Im Verlauf entwickelt sich zusätzlich eine rechtsseitige Hemiparese mit dem Kraftgrad (nach Medical Research Council) 3–4/5, sodass sich der NIHSS-Score auf 7 erhöht. In den Laboruntersuchungen bei Aufnahme sind die Thrombozytenzahl mit 215 × 10^3^/µl, die partielle Thromboplastinzeit (PTT) mit 37,1 s und die International Normalized Ratio (INR) mit 1,06 normwertig.

### Diagnose

**Figure 1:**
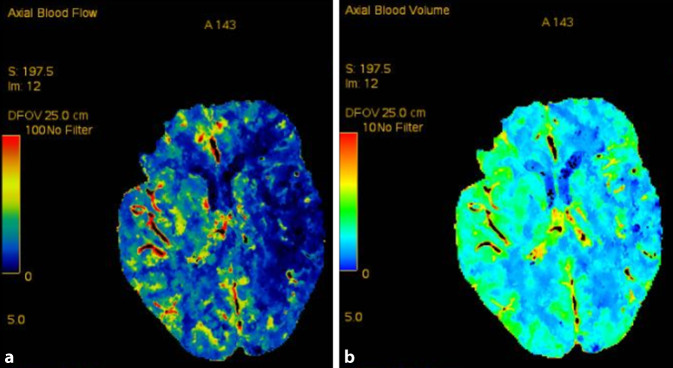

In der computertomographischen Angiographie (CTA) stellt sich ein Verschluss der linken A. cerebri media im M2-Segment mit einem ausgedehnten Perfusionsdefizit noch ohne Nekrose („mismatch“) dar (Abb. 1). Schon in der initialen CT finden sich in beiden Lungenspitzen milchglasartige Trübungen. Die SARS-CoV-2-Infektion kann mithilfe der „polymerase chain reaction“ (PCR) gesichert werden.

### Therapie und Verlauf

**Figure 2:**
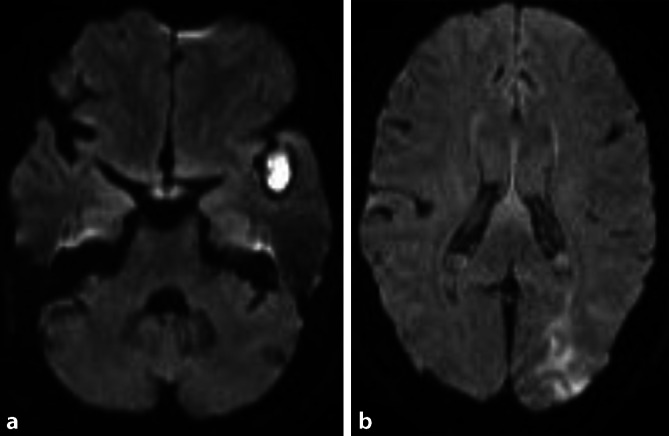

Es erfolgt eine systemische Thrombolyse mit rekombinantem gewebsspezifischem Plasminogenaktivator (rt-PA, 0,9 mg/kgKG) und anschließender mechanischer Thrombektomie. In der Magnetresonanztomographie (MRT) zeigt sich eine sekundäre Einblutung links-temporal ohne Nachweis einer territorialen größeren Infarktdemarkation (Abb. 2). Zudem entwickelt sich eine schwere COVID-19-Pneumonie mit bakterieller Superinfektion, sodass eine invasive Beatmung und empirische Therapie zunächst mit Ceftriaxon und dann mit Piperacillin+Tazobactam erfolgt. Nach 12 Tagen kann die Patientin extubiert und auf die neurologische Allgemeinstation verlegt werden. Es ergeben sich keine Hinweise auf eine kardioembolische Ursache im Langzeit-EKG, in der transthorakalen (TTE) und transösophagealen Echokardiographie (TEE) sowie ebenso wenig Hinweise auf eine arterio-arteriell-embolische Genese in der CTA und extra- und transkraniellen Duplexsonographie der hirnversorgenden Arterien. Nach insgesamt 4 Wochen klinischem Aufenthalt wird die Patientin mit einer leichtgradigen rechtsseitigen Hemiparese in die neurologische Rehabilitation verlegt.

## Fall 2

### Anamnese

Ein 56-jähriger Patient mit einer vor 9 Tagen in der PCR bestätigten SARS-CoV-2-Infektion stellt sich mit dem Rettungsdienst wegen einer Verschlechterung des Allgemeinzustands sowie Dyspnoe und Fieber vor. Aus der Vorgeschichte sind ein allergisches Asthma und eine Katzenhaarallergie bekannt, für die er Salbutamol sowie Fenoterol und Ipratropiumbromid als Spray und einzige Medikation einnimmt.

### Befund

Auskultatorisch ist ubiquitär ein abgeschwächtes Atemgeräusch mit beidseitigem Knistern zu verzeichnen. Die Blutgasanalyse weist mit einer Sauerstoffsättigung von 75 % unter Maskenbeatmung mit 6 l Sauerstoff/min hin auf eine respiratorische Insuffizienz vom hypoxämischen Typ hin. Bei der Aufnahme betragen die Thrombozytenzahlen 153 Gpt/µl, die INR 1,04 und die PTT 37,1 s; die Konzentration der D-Dimere ist mit 1,52 µg/nl erhöht (Referenzbereich 0–0,5 µg/nl).

### Diagnose

**Figure 3:**
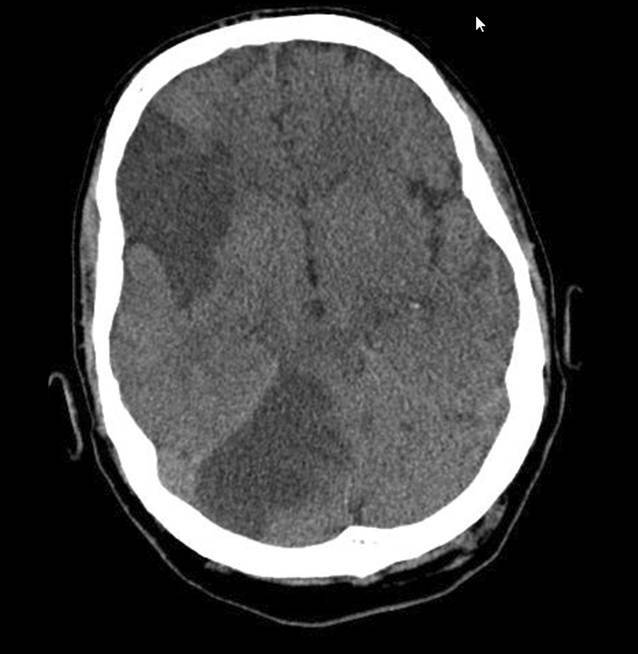

Im Thorax-CT finden sich passend zu einer COVID-19-Pneumonie bilateral diffuse entzündlich-infiltrative Milchglastrübungen mit positivem Bronchopneumogramm und Segmentembolien im linken Unterlappen. Im CT des Abdomens stellt sich ein Milzinfarkt dar. Eine Schädel-CT zeigt im Verlauf am 8. Behandlungstag demarkierte subakute Infarkte in der rechten Kleinhirnhemisphäre und im Stromgebiet der rechten A. cerebri media (Abb. 3).

### Therapie und Verlauf

Bei dem Patienten treten 4 Tage nach der Krankenhausaufnahme unter nichtinvasiver Beatmung ein zunehmendes respiratorisches Versagen und Ängste auf, sodass er endotracheal intubiert und invasiv beatmet werden muss. Wegen einer ausgeprägten bronchopulmonalen Spastik werden Budesonid und Reproterol über einen Perfusor verabreicht. Seit der stationären Aufnahme (d. h. vor der Diagnose der Lungenarterienembolien) wird er mit unfraktioniertem Heparin behandelt (gesteuert mithilfe der PTT, Zielwert: 50 s). Im Verlauf wird unter dem Verdacht auf eine Heparin-induzierte Thrombozytopenie vom Typ II auf Argatroban umgestellt. Wegen geminderter Erweckbarkeit erfolgt die oben genannte CT des Kopfes, in der sich die Hirninfarkte darstellen (eine Verlaufsuntersuchung erfolgt wegen des zunehmenden Lungenversagens nicht). Mithilfe der TEE wird ein großes persistierendes Foramen ovale (PFO) festgestellt; eine Ultraschalluntersuchung der Beinvenen wird aufgrund fehlender klinischer Thrombosezeichen nicht durchgeführt. Der klinische Zustand des Patienten verschlechtert sich mit zunehmender Gasaustauschstörung und bipulmonalen Verdichtungen im Sinne eines „adult respiratory distress syndrome“, an dem er am 13. Tag der Intensivbehandlung verstirbt. Die Zustimmung zur Obduktion wird nicht erteilt.

## Diskussion

Beide Patienten erlitten im mittleren Alter bei wenigen Vorerkrankungen einen Verschluss großer Hirngefäße im Rahmen der COVID-19. Im ersten Fall waren zum Zeitpunkt des Hirninfarkts noch keine respiratorischen Symptome aufgetreten, während diese beim zweiten Patienten den Hirninfarkten vorangingen. Die erste Patientin wies in der Diagnostik keine Hinweise auf eine kardiale oder arterielle Emboliequelle auf, während bei dem zweiten Patienten mit dem PFO eine wahrscheinliche Ursache für paradoxe Embolien aus dem venösen System vorlag. Etwa 1,1–1,4 % der Betroffenen einer (diagnostizierten) SARS-CoV-2-Infektion erleiden einen Schlaganfall [6, 8]. Zwar ist im Einzelfall ein kausaler Zusammenhang zwischen COVID-19 und dem Eintreten eines Hirninfarkts nicht beweisbar, in einer retrospektiven Kohortenstudie [9] wurde aber ein erhöhtes Risiko für Hirninfarkte von COVID-19-Patienten im Vergleich zu nicht an COVID-19 Erkrankten von 30 % ermittelt.

Etwa 1,1–1,4 % der Patienten mit (diagnostizierter) SARS-CoV-2-Infektion erleiden einen Schlaganfall

Erhöht ist nach einer rezenten Metaanalyse [6] im Vergleich zu Schlaganfällen ohne COVID-19 auch das Risiko für einen Verschluss großer Hirnarterien („odds ratio“ [OR] 2,73), für einen erhöhten NIHSS-Score (Median: 5 Punkte) im Sinne eines größeren neurologischen Defizits und für eine erhöhte Krankenhausmortalität (OR 5,21). Zudem waren die Patienten durchschnittlich 6 Jahre jünger und wiesen weniger kardiovaskuläre Risikofaktoren auf als Schlaganfallpatienten ohne COVID-19 [6]. In der Literatur (Metaanalyse: [10]) werden bei COVID-19 sowohl eine Koagulopathie mit erhöhten Konzentrationen der D‑Dimere als auch Vaskulitiden und virale Kardiomyopathien als mögliche Ursachen diskutiert; die Häufigkeit von Schlaganfällen ungeklärter Ursache ist bei COVID-19-Patienten erhöht (d. h. ohne identifizierte makroangiopathische, mikroangiopathische oder kardiale Ursache, OR 3,4, [6]).

Die Häufigkeit von Schlaganfällen ungeklärter Ursache ist bei COVID-19-Patienten erhöht

Der zweite Patient erlitt trotz antithrombotischer Prophylaxe mit PTT-gesteuerter Infusion von unfraktioniertem Heparin Lungenarterienembolien und bei vorliegendem PFO multiple Hirn- und Milzinfarkte, deren Ursache venöse Thrombosen bei einer COVID-19-induzierten Koagulopathie gewesen sein könnten – klinische Zeichen von Beinvenenthrombosen lagen nicht vor. Bei der ersten Patientin kommt eine endotheliale Schädigung bei COVID-19 mit sekundärer hämorrhagischer Transformation infrage [10]. Bezüglich des Outcome verwirklichte sich im zweiten Fall die häufige eintretende ungünstige Prognose von Hirninfarkten bei COVID-19 [2–4, 6]. Die erfolgreiche systemische Thrombolyse, gefolgt von einer mechanischen Thrombektomie, bei der ersten Patientin macht dennoch deutlich, dass die etablierten Therapiealgorithmen [1, 5] der Behandlung ischämischer Schlaganfälle auch bei diesen Patienten anwendbar sind. In Zukunft wird die Klärung der Ätiologie ischämischer Ereignisse bei COVID-19-Patienten für die Entwicklung von prophylaktischen und weiteren Therapieoptionen bedeutsam sein.

## Fazit für die Praxis

- Ein zerebrovaskuläres Ereignis ist keine seltene Komplikation im Rahmen einer Coronavirus-19-Erkrankung (COVID-19) und geht häufig mit einem schlechten Outcome einher.
- Zeichen einer gestörten Gerinnung sowie neu auftretende neurologische Symptome sollten rasch erkannt und behandelt werden.
- Eine umgehend eingeleitete leitliniengerechte Schlaganfalltherapie mit i.v.-Thrombolyse und mechanischer Thrombektomie kann die Prognose auch bei Patienten mit COVID-19 günstig beeinflussen.
- Zur Genese und zur optimalen Behandlung der Koagulopathie bei COVID-19 besteht weiterer Forschungsbedarf.
